# The visual corticostriatal loop through the tail of the caudate: circuitry and function

**DOI:** 10.3389/fnsys.2013.00104

**Published:** 2013-12-06

**Authors:** Carol A. Seger

**Affiliations:** Program in Molecular, Cellular, and Integrative Neuroscience, Department of Psychology, Colorado State UniversityFort Collins, CO, USA

**Keywords:** striatum, caudate, category learning, basal ganglia, corticostriatal, recurrent neural network, reinforcement learning, Area TE

## Abstract

Although high level visual cortex projects to a specific region of the striatum, the tail of the caudate, and participates in corticostriatal loops, the function of this visual corticostriatal system is not well understood. This article first reviews what is known about the anatomy of the visual corticostriatal loop across mammals, including rodents, cats, monkeys, and humans. Like other corticostriatal systems, the visual corticostriatal system includes both closed loop components (recurrent projections that return to the originating cortical location) and open loop components (projections that terminate in other neural regions). The article then reviews what previous empirical research has shown about the function of the tail of the caudate. The article finally addresses the possible functions of the closed and open loop connections of the visual loop in the context of theories and computational models of corticostriatal function.

## Introduction

Modern research in the basal ganglia has become increasingly focused on cognitive functions, an extension from early work that focused on the role of dorsal circuits in motor processing, and ventral circuits through the nucleus accumbens in reward and addiction. However, within the domain of cognition, researchers have concentrated on interactions between the prefrontal cortex and anterior regions of the striatum underlying executive functions. The interactions of temporal lobe cortex with the posterior striatum, specifically the tail of the caudate nucleus, have been minimally studied. This is likely due to a combination of factors, including the lack of a good rodent model for this system, and methodological difficulties in accessing, isolating, and measuring activity in the tail of the caudate. However, in recent years there has been a significant increase in research on the visual corticostriatal loop, which may signal that this structure's time has come. This goal of this paper is to provide a thorough review of the anatomy and function of the visual corticostriatal loop. It first provides a detailed review of what is known (and not known) about the corticostriatal circuitry passing through the tail of the caudate, and summarizes empirical studies investigating tail of the caudate function. It then surveys computational neuroscience to explore potential functions of these circuits, and proposes several future directions for research.

## Anatomy of the visual corticostriatal system

The visual corticostriatal loop consists of the lateral and inferior temporal higher order visual cortex, its target regions in the tail and genu of the caudate, and subsequent projections through basal ganglia output nuclei to thalamus. This section traces this circuitry, beginning with the anatomy of the tail of the caudate, then describing the projections from visual cortex to caudate, caudate to substantia nigra pars reticulata (SNr), and finally from SNr to thalamus and back to cortex, or to superior colliculus, forming both open and closed loops. The focus is on the primate brain, both human and macaque monkey, though other species including rat and cat are also discussed where relevant studies are available. The focus is also on higher order visual projections, but because the temporal auditory cortex also projects to adjacent regions of the posterior caudate it will be discussed where relevant.

### The tail of the caudate

**Figure 1:**
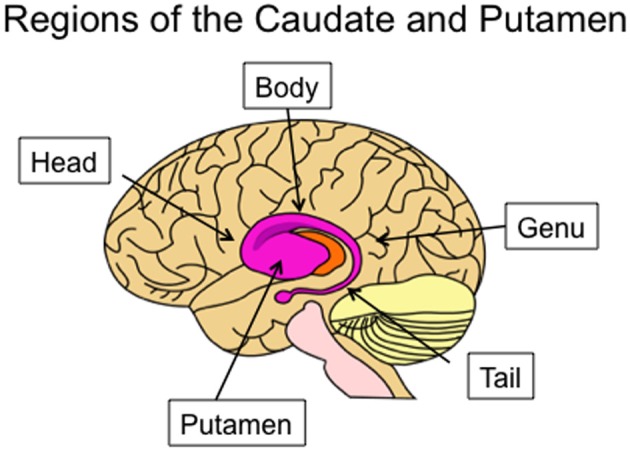
**Lateral view of the human caudate and putamen, situated within a transparent brain**. The subregions of the caudate are indicated: the head, body, genu, and tail.

**Figure 2:**
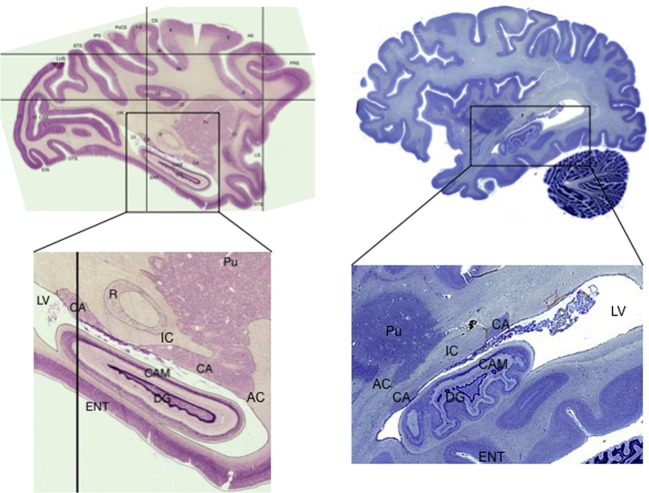
**Sagittal sections showing the tail of the caudate in macaque monkey (*Macaca mulatta*) and human**. Note the narrow width of the structure, how it follows along the lateral ventricle, and how it passes adjacent to the hippocampus. Macaque images from BrainMaps: An Interactive Multiresolution Brain Atlas; http://brainmaps.org [retrieved on 9-24-2013]. Human images from the Michigan State University Brain Biodiversity Bank https://www.msu.edu/~brains/brains/human/index.html [retrieved on 9-24-2013]. CA: Caudate nucleus, tail. Pu: Putamen. IC: Internal Capsule. AC: Anterior Commissure. DG: Dentate Gyrus CAM: Cornu Ammonis (hippocampus). ENT: Entorhinal cortex. LV: Lateral Ventricle. R: red nucleus (not shown in human).

The tail of the caudate nucleus is a subregion of the basal ganglia. Overall, the basal ganglia consist of three subcortical nuclei: the caudate, putamen, and globus pallidus. The caudate and putamen, together, are collectively referred to as the striatum. The caudate nucleus is located immediately lateral to the ventricles, and has a rather unusual spiral shape, as illustrated in Figure 1. The largest portion of the caudate is the anterior and medial region, which is referred to as the head. From the head, the caudate extends in posterior and lateral direction through the body, turns in an inferior direction through the genu, and finally projects in an anterior direction through the tail. As illustrated in Figure 2, the anterior portion of the tail of the caudate passes through the medial temporal lobe. It runs superior to the hippocampus, divided from this structure by only a narrow portion of the lateral ventricle. From posterior to anterior, the tail also passes close by the fimbria, immediately lateral to the lateral geniculate, inferior to the putamen, and finally adjacent to the amygdala. It is medial to deep portions of the middle temporal cortex, and dorsomedial to medial temporal cortex regions including the entorhinal cortex. Many of these adjacent structures are also associated with learning and memory, which provides many challenges for dissociating the functions of these structures. These challenges are further discussed in sections Human Neuroimaging and Lesion Studies below.

In terms of chemical neuroanatomy, the tail and body of the caudate have higher levels of cholinergic interneurons than other regions of the striatum (Bernácer et al., 2007). Dopamine projections to the striatum overall show a gradient from highest density in the more antero-medial-inferior regions, out to the more poster-lateral-superior regions; the tail of the caudate thus appears likely to have similar dopamine projections as other posterolateral regions such as the body of the caudate and posterior putamen (Haber et al., 2006).

### Projections from visual cortex to the caudate tail

#### General characteristics of the corticostriatal system: loops and projections

**Figure 3:**
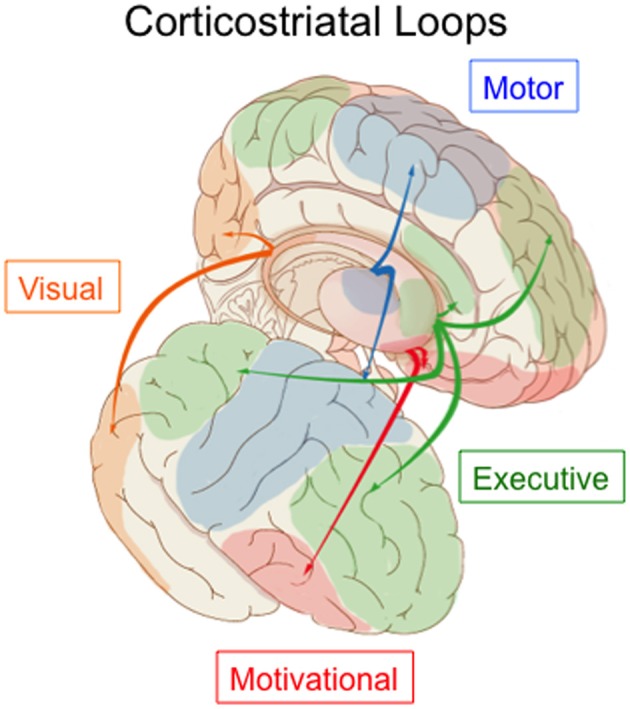
**Parcellation of the corticostriatal system into four loops**. The visual loop, indicated in orange, connects extrastriate and inferior temporal visual cortex to the tail of the caudate nucleus. Figure based on information in Lawrence et al. (1998) and Seger (2008).

The dominant view, since the classic paper by Alexander et al. (1986), has been that the corticostriatal system is structured as a set of independent recurrent loops. The primary loop structure includes projections from the cortex, to the striatum (caudate and putamen), to the globus pallidus and/or SNr, to the thalamus, and finally back to cortex. Tract tracing methods revealed that cortical regions projected topographically, such that different cortical regions projected to different parts of the striatum. Overall the corticostriatal projections form a continuous system with projections from medial-anterior regions of cortex (e.g., orbitofrontal cortex) out to lateral-posterior regions (e.g., superior parietal cortex) generally projecting along a ventro-anterior-medial (e.g., nucleus accumbens) to dorso-posterior-lateral (e.g., posterior putamen) gradient in the striatum. For heuristic purposes, this system has been divided into separate loops in order to highlight different functions, but any such division is ultimately arbitrary. The most common, and widely accepted, division is into three loops: limbic through the ventral striatum, motor through the middle and posterior putamen, and associative through the anterior striatum (including the head of the caudate and anterior putamen). This division includes the visual projections from temporal cortex in the associative loop. However, in primates projections from prefrontal regions differ from those from temporal cortex, with the latter projecting to more posterior caudate and putamen. Lawrence et al. (1998) recognized this and proposed a division into four basic loops, illustrated in Figure 3, including the visual loop as a separate network.

These four loops have additional meaningful subdivisions. For example, premotor and primary motor regions interact with different regions of the putamen. One additional loop that is particularly relevant for understanding the visual loop is the oculomotor loop (normally considered part of the executive loop, though it is similar functionally to parts of the motor loop as well). The oculomotor loop connects cortical regions involved in visual attention and eye movement planning to the body of the caudate nucleus. This region of the caudate has been shown to be sensitive to visual information as well (Ding and Gold, 2013; Watanabe and Munoz, 2013). The possible interactions of the oculomotor and visual loops are discussed further in section Visual Attention and Eye Movement Control below.

At the cellular level, individual corticostriatal projection neurons typically make multiple (though sparse) synapses on multiple striatal spiny neurons along an axon that projects longitudinally across the caudate and putamen in a roughly anterior to posterior direction (Selemon and Goldman-Rakic, 1985). As a result, within the visual loop, large scale projection zones from regions of cortex also tend to be oriented longitudinally along the caudate, and are often so narrow so that they do not extend across the entire width of the tail of the caudate but rather are localized to a lateral, medial, dorsal, or ventral portion (as indicated in Table 1 for monkey tracer studies).

**Table 1:** **Tracer studies examining projections from extrastriate visual cortex to striatum in monkey**.

**Temporal region**	**Posterior caudate**	**Posterior putamen**	**Anterior striatum**	**Tracer**	**Paper**	**Injection or Figure**
**TEMPORAL POLE**						
Temporal pole	Tail, medial	–	Medial	[3H]-AA	Van Hoesen et al., 1981	1, 2
	Tail	Ventral	Widespread ventral regions	[3H]-AA	Yeterian and Pandya, 1998	5, 8
**AREA TE (LATERAL PORTION)**						
TE	Tail, lateral	Ventrolateral	–	[3H]-AA	Van Hoesen et al., 1981	5, 6
TE	Tail and genu	Ventral	Head-body junction	WPA-HRP	Baizer et al., 1993	6
TE-dorsal	Tail, lateral	Lateral	–	PVL	Cheng et al., 1997	
TE	Tail	Ventral	Head-body junction	WPA-HRP, [3H]-AA	Webster et al., 1993	All
Squirrel monkey ITr	Tail, lateral	Ventral	Middle head	Various	Steele and Weller, 1993	
TE-dorsal	Tail	Posterior	Body	[3H]-AA	Yeterian and Pandya, 1995	18
**AREA TE (VENTRAL PORTION)**						
TE-ventral	Tail	Posterior	Head	[3H]-AA	Yeterian and Pandya, 1995	17
TE-ventral	Tail and genu, lateral	Posterior, lateral	Head, anterior putamen	[3H]-AA	Van Hoesen et al., 1981	7, 8, 9
TE-ventral	Tail, lateral	Lateral	Ventral striatum	PVL	Cheng et al., 1997	
**AREA TEO (POSTERIOR TO TE)**						
Squirrel monkey ITc	Tail and genu	Ventral	Middle head	Various	Steele and Weller, 1993	
TEO-lateral	Tail and genu	Ventral	Head and body	WPA-HRP + [3H]-AA	Webster et al., 1993	All
TEO-medial	Tail	Small posterior	Superior head and body	[3H]-AA	Yeterian and Pandya, 1995	16
TEO-intermediate	Tail and genu	Posterior	Superior head and ventral body	[3H]-AA	Yeterian and Pandya, 1995	14
**SUPERIOR TEMPORAL–ANTERIOR (TA)**						
TA	–	Ventral	Head	[3H]-AA	Van Hoesen et al., 1981	3, 4
TA	Tail	–	Middle and ventral head	[3H]-AA	Yeterian and Pandya, 1998	10, 11
**SUPERIOR TEMPORAL–POSTERIOR TO TA**						
Posterior superior temporal	Tail and ventral body	Ventral	Anterior putamen and caudate	[3H]-AA	Yeterian and Pandya, 1998	1, 2, 4
**SUPERIOR TEMPORAL–FAR POSTERIOR**						
MT	Tail, genu, and body	Posterior	–	[3H]-AA	Maioli et al., 1983	All
	Body	Superior and posterior	Head and body	[3H]-AA	Yeterian and Pandya, 1998	14, 15
	Tail, genu and body	Posterior	Head-body junction	[3H]-AA	Yeterian and Pandya, 1995	8, 10, 12

[3H]-AA: radiolabeled amino acids, typically a mixture of [3H]-proline and [3H]-leucine. WPA-HRP: Wheat germ agglutinin conjugated to horseradish peroxidase. PVL: Phaseolus vulgaris leucoagglutinin.

The degree of convergence of cortical projection neurons on striatal neurons has been controversial. Some theories argued for broad convergence, so that axons from different cortical regions converge on the same striatal neurons. Later cellular based studies shed doubt on that view (see Bar-Gad et al., 2003 for review). First, although tracer studies often find large projection zones, more fine grained tracer studies show that within these zones the projections are not evenly distributed, but rather have “patchy” patterns of innervation in which some subareas within the overall region are innervated and others are not (Goldman and Nauta, 1977). Although individual projection neurons can extend a considerable distance along the striatum, they make very sparse synapses, which reduces numerically the potential for convergence (Zheng and Wilson, 2002). Each striatal neuron receives input from at most 0.01% of the corticostriatal projection neurons (Bar-Gad et al., 2003), and adjacent striatal neurons likely do not share cortical afferents (Zheng and Wilson, 2002). When there is convergence, the likelihood is high that the input is from nearby cortical neurons (Kincaid et al., 1998).

#### The corticostriatal projection from visual temporal cortex in non-human mammals

An early influential identification of corticostriatal projections from temporal cortex to the tail of the caudate came from a study that examined axonal degeneration after ablation of striatal tissue (Kemp and Powell, 1970). More detailed information came from later studies using anterograde and retrograde tracers. The results of studies examining projections from the visual and auditory regions of the temporal lobe using anterograde tracers in monkeys are summarized in Table 1. In general, the target structures in the striatum of visual temporal cortex fall in three regions. First is the tail of the caudate nucleus, sometimes extending into the genu and body of the caudate. Second is the posterior putamen, which is adjacent to the tail of the caudate. Third is a discontinuous region of the dorsal head and/or body of the caudate. Relatively anterior temporal regions (e.g., region TE, Table 1) tend to project to areas further down the tail than more posterior temporal regions (e.g., region TEO, Table 1). These studies are complemented by a study (Saint-Cyr et al., 1990) that applied a retrograde tracer in the tail and genu of the caudate and found that it received broad projections from temporal visual regions, with relatively posterior regions projecting to the genu and relatively anterior to the tail. Little is known about convergence of extrastriate visual regions. As shown in Table 1, the different temporal regions project to similar but not identical striatal territories. However, only one study has examined individual axons projecting from higher order visual cortex to the tail of the caudate (Cheng et al., 1997). It found that convergence onto striatal modules was limited to input from cells within the same or adjacent cortical columns. Cheng et al. argued that these projections represent related but not identical features of an object and could be useful in forming an integrated visual representation.

Cats have well developed visual systems and served as model species in most early visual electrophysiological studies. Updyke (1993) examined subcortical connections between 11 extrastriate visual areas commonly studied in electrophysiological studies. He found results broadly consistent with tracer studies in monkeys: visual cortex projected to longitudinal territories within the caudate nucleus that extended from the dorsal head, through the body, and into the tail. Visual cortex also projected to the posterolateral putamen.

Visual projections have also been studied in rodents, though there are significant differences from primates. Rodents have very limited cortical vision compared with primates (Baker, 2013), and the gross anatomy of the rodent striatum is significantly different. In rodent striatum, the anterior caudate and putamen form a single structure, usually divided into dorsomedial and dorsolateral striatum, and the posterior region of the striatum differs in shape from the primate tail of the caudate, which makes it difficult to establish clear homology. Visual cortex in the rodent is on lateral and posterior cortical surface, adjacent to auditory cortex. Faull and colleagues (Faull et al., 1986; McGeorge and Faull, 1989) placed retrograde tracers in multiple striatal regions, and found that auditory cortex projected to the most ventral and caudal region of the striatum, and visual cortex to an adjoining but more rostral region within the dorsomedial striatum. More recent research has argued that association area projections, including those from visual and auditory cortex, also project to a distinct dorsocentral region of striatum, and may converge with regions of parietal cortex important for the control of spatial attention (Cheatwood et al., 2003, 2005; Reep et al., 2003). There is evidence that primary visual cortex in the rodent also projects to the striatum, in contrast to absence of such projections in monkey (López-Figueroa et al., 1995).

An alternative approach to mapping projections from cortex to striatum is through direct activation or deactivation of one region combined with a measure of activity from the other region. Glynn and Ahmad (2002) directly stimulated individual cortical regions in rats while recording from multiple striatal regions. Stimulation of secondary visual regions in the occipital lobe lead to greatest activity in posterior and slightly anterior medial regions, whereas stimulation of auditory regions led to strong posterior activity. These results are consistent with the tracer studies performed by Faull and colleagues summarized above. Cohen (1972) found that stimulating the inferior temporal cortex or tail of the caudate in the monkey had similar effects on discrimination learning, and these effects differed from when stimulation was presented to the dorsolateral prefrontal cortex or anterior striatum.

#### Visual corticostriatal projections in humans

There has been little examination of the visual corticostriatal projection in humans. Anatomical imaging studies using diffusion tensor imaging (and related techniques) have typically focused on projections from the frontal cortex and have not reported connections with the temporal cortex (Draganski et al., 2008; Verstynen et al., 2012). One early study, although limited (it compared the entire caudate to the entire putamen), did report substantial temporal cortex connections with the caudate nucleus (Leh et al., 2007). Projections from temporal cortex to posterior caudate are understudied for two reasons: first, projections from temporal lobe to basal ganglia are hard to follow using current DTI methods because of twists or kinks in the pathways, and second, as is discussed in more detail in section Human Neuroimaging below, the atlases commonly used in neuroimaging do not include the tail of the caudate.

A new approach is utilizing resting state fMRI to identify circuits with intrinsic connectivity. Several recent studies have shown that resting state fMRI has a good correspondence with known anatomical connections; however, it should be noted that resting state cannot tell us which regions are directly connected, but rather just tells us which regions tend to coactivate (Hermundstad et al., 2013). Choi et al. (2012) examined connectivity between known cortical networks and the basal ganglia. As predicted from the anatomical connections, resting state networks that include the inferotemporal cortex were shown to correlate with relatively posterior regions of the caudate nucleus. One important caveat is that the caudate region examined did not include the tail of the caudate and only extended through part of the body of the caudate, and therefore is likely to underreport or completely miss connectivity with visual cortex.

### Pathways through the basal ganglia output nuclei

After the striatum, information passes through the basal ganglia output nuclei, including the globus pallidus, both internal (GPi) and external (GPe) portions, and the SNr. There are two primary pathways, which are termed the direct and indirect pathways. The direct pathway involves projections from striatum directly to SNr or GPi. The indirect pathway passes first to the GPe, and then to the SNr or GPi. In the primary visual loop, direct pathway projections target a lateral portion of the SNr (Saint-Cyr et al., 1990; Middleton and Strick, 1996; Maurin et al., 1999). The lateral SNr is also a target of auditory projections (Kolomiets et al., 2003). In the rodent, auditory and visual projections also target the SNr, but they extend to ventral as well as lateral subregions (Faull et al., 1986).

The motor and executive corticostriatal loops also have a third pathway, termed the hyperdirect pathway, that projects to the sub thalamic nucleus (STN) rather than the striatum, and from there to the SNr/GPi. Studies have shown that associative prefrontal regions as well as motor regions project to the STN in a topographic manner in primates (Mathai and Smith, 2011; Haynes and Haber, 2013). However, only one study has explicitly examined whether temporal cortex projects to the STN; it found no projections from a posterior superior temporal region (Afsharpour, 1985). Researchers assume therefore that there is no visual hyperdirect pathway, though Coizet et al. (2009) argue that the STN may receive visual information indirectly, but nevertheless rapidly, from the superior colliculus.

All three pathways converge onto a common inhibitory projection to the thalamus. The subsequent projections from thalamus to cortex are excitatory, and therefore the net tonic effect of this inhibitory projection onto the thalamus is to keep activity levels low in both thalamus and cortex. The direct pathway phasically releases the thalamus from inhibition and allows excitatory output to cortex; the indirect and hyperdirect pathways increase the inhibition of thalamus and cortex across different time scales cortex (DeLong, 1990; Mink, 1996; Frank, 2005).

### Closing the loop: projections to thalamus and back to cortex

**Figure 4:**
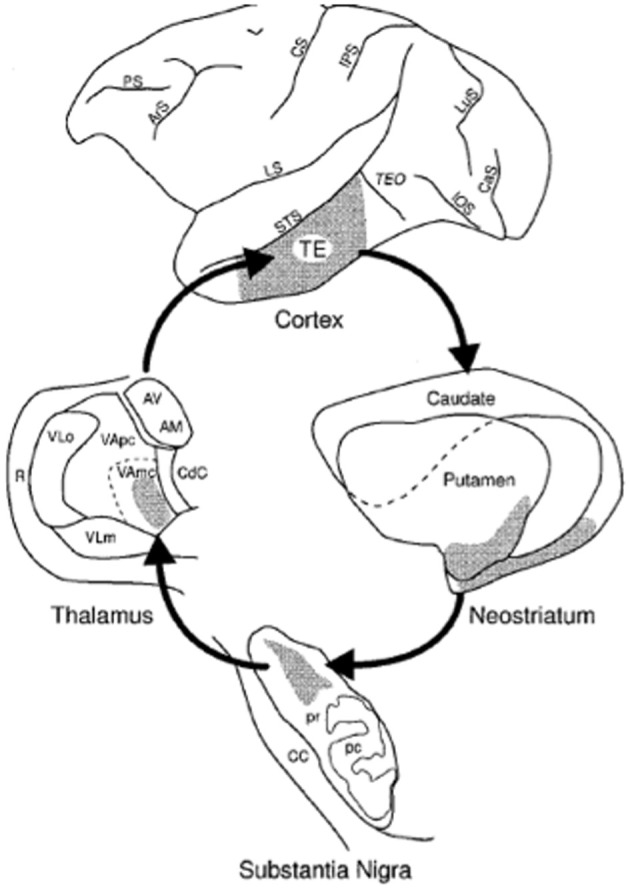
**Illustration from Middleton and Strick (1996 Figure 4) showing the closed loop visual corticostriatal connections they found using multisynaptic rabies virus tracing**.

The canonical closed loop of the basal ganglia involves a return projection from the thalamus to the originating area of cortex. Tracing out the entire loop, and in particular the return projections from thalamus, is very difficult to do with tracer studies and typically requires the use of viruses that can map out multisynaptic pathways. The only study that has done this for the visual loop is Middleton and Strick (1996) who traced projections from the SNr to thalamus area VAmc to temporal lobe area TE, complementing the research finding projections from TE to basal ganglia. This closed loop is illustrated in Figure 4. In their subsequent review article Middleton and Strick (2000) mention research finding recurrent connections to parietal cortex (Clower et al., 2005), and conclude that while it is still unknown whether all the cortical areas that target BG also receive connections from BG, closed loop circuits may be a fundamental feature of the basal ganglia.

### Open loops from the visual corticostriatal system

**Figure 5:**
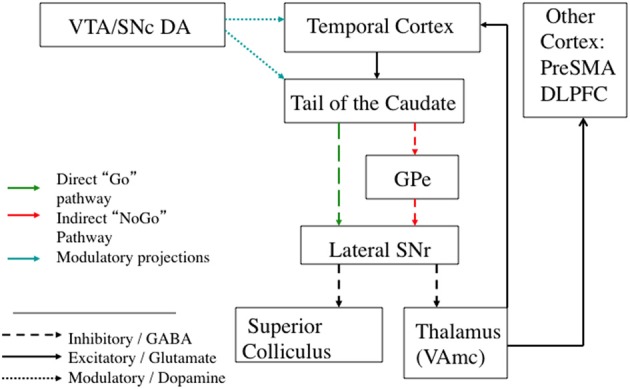
**Schematic diagram of the visual corticostriatal loop, including the best established open and closed loop connections**. Broad regions of extrastriate and visual temporal cortex project to the tail of the caudate. From the tail of the caudate, direct pathway projections go to the lateral SNr. There are open loop projections from SNr to superior colliculus that can directly release eye movements. There are also projections to the thalamus. From thalamus, there are projections back to cortex that close the loop, as well as open loop projections to other cortical regions, such as the pre-SMA. The hyperdirect pathway is not included because it is unknown if visual cortex projects to STN; see text for details. GPe: Globus Pallidus, external portion. Pre-SMA: Pre supplementary motor area. DLPFC: Dorsolateral Prefrontal Cortex. SNr: Substantia Nigra pars reticulata. VTA: Ventral Tegmental Area. SNc: Substantia Nigra pars compacta.

The basal ganglia also have a number of different open loop projections (Lopez-Paniagua and Seger, 2011); the primary ones are diagrammed along with the closed loop in Figure 5. An open loop projection is one that targets a different structure than the originating cortical region (Joel and Weiner, 1994). One well established open loop projection is to the superior colliculus, and allows visual information processed in the visual loop to directly elicit saccadic eye movements (Hikosaka et al., 2013).

Another group of open loop projections pass through the thalamus. VAmc, the region of the thalamus that receives visual loop projections, projects to more than just visual cortex. One important target of VAmc is the pre-supplementary motor area (pre-SMA) (Nakano et al., 1992), an area involved in processes integrating higher order motor control with executive functions in the dorsolateral prefrontal cortex. The COVIS model of visual categorization learning is based on this open loop connection between the visual loop and pre-SMA, and can successfully account for many aspects of learning (Ashby et al., 1998, 2007). The thalamus also makes direct projections to striatum, and information from the thalamus may therefore also affect other corticostriatal loops without returning to cortex (Joel and Weiner, 1994; McFarland and Haber, 2000).

Another type of corticostriatal loop involves direct projections from the SNr to striatum, bypassing the return projections through the thalamus and cortex. This projection has been shown to have both recurrent closed-loop aspects and open-loop aspects (Haber et al., 2000). Haber and colleagues refer to these projections as forming an “ascending spiral” because the open loop projections tend to target the striatal regions at the next step along the overall antero-medial-inferior to postero-lateral-superior gradient from motivational to associative to motor loops. These projections also exist in rodents, with return SNr connections both proximal to the originating region, as well as to more distal associative regions (Maurin et al., 1999; Mailly et al., 2003). These connections have been verified in the cat as well (Harting et al., 2001).

## Empirical research examining the functions of the caudate tail

This section surveys what is known about the function of the tail of the caudate from neuroimaging, neurophysiological, and lesion studies. There are relatively few studies that specifically target the tail of the caudate activity, especially in contrast to the number of studies examining the head of the caudate or putamen. One reason may be the experimental challenges posed by the unusual shape and location of the tail of the caudate for neuroimaging and lesion research, which are discussed in more detail in each section below.

### Human neuroimaging

**Figure 6:**
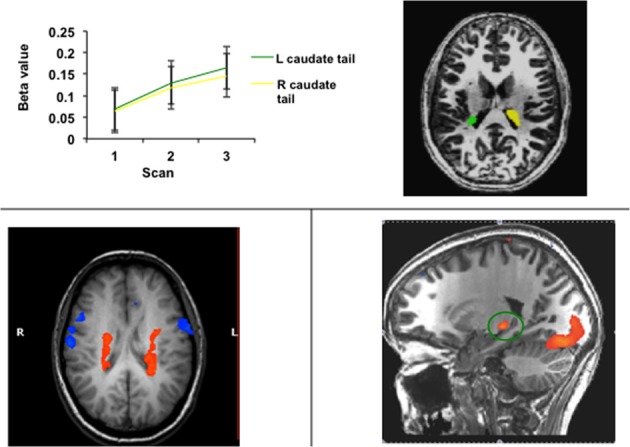
**Activity in the tail of the caudate nucleus in functional imaging studies of categorization and related learning tasks**. (Top) Regions of the right and left caudate tail that increased in activity across blocks of learning; data from Seger et al. (2010) (Bottom left) Bilateral region of the body and tail of the caudate active during learning of both probabilistic and deterministic stimulus-category relationships. Data adapted from Seger and Cincotta (2005). (Bottom right) Region of the caudate tail (green circle) that was more active during the stimulus-response portion of categorization trials than during the feedback receipt portion; data from Lopez-Paniagua and Seger (2011).

Researchers in the fields of category learning and visual classification learning have targeted the tail of the caudate, inspired by theories of visual categorization proposing that the open loop projection from the visual loop to premotor cortex could serve as a plausible biological substrate for incremental and implicit category learning (Ashby et al., 1998; Seger, 2008). The tail and adjoining regions of the body of the caudate are recruited in these tasks, as illustrated in Figure 6. Activity often follows the time course of learning, increasing as accuracy continues to increase (Seger et al., 2010). Activity also correlates with learning, such that subjects who learn better have higher recruitment in this region (Seger and Cincotta, 2005, 2006), and activity is higher for correctly categorized trials than error trials (Nomura et al., 2007). The tail of the caudate is recruited across a variety of different category learning tasks, including rule based tasks, and information integration tasks, and for both deterministic and probabilistic stimulus—category relationships (Seger and Cincotta, 2005), indicating it is not dependent on a particular stimulus type or category structure (Seger, 2008). Activity is present both when subjects are learning via trial and error, and when learning via observation (Cincotta and Seger, 2007), and is greater at the time of stimulus-response processing than at the time of feedback receipt (Lopez-Paniagua and Seger, 2011).

Human neuroimaging typically is performed on a whole brain basis. However, for several reasons tail of the caudate activity can easily be missed. One reason is limitations in the normalization algorithms, that typically are optimized to maximize accuracy for cortical rather than subcortical structures. Without precise normalization, activity in the tail of the caudate in individuals will not overlap in a group analysis, and no apparent group activity will be detected. A second reason is that standard neuroimaging atlases such as the Harvard-Oxford structural atlas used with neuroimaging analysis programs such as FreeSurfer truncate the caudate at the body, and completely exclude the tail. Many studies use these ROIs as a template for spatial normalization, for region of interest based analyses, or for small volume correction for multiple comparisons. A final reason is that the tail of the caudate is close to the hippocampus, and could be misidentified as such especially in tasks involving learning and memory. Therefore, the tail of the caudate may be recruited in additional cognitive tasks, but yet not have been properly identified and reported in the neuroimaging literature. Future work should use high resolution scanning and may need to modulate parameters to maximize potential signal. One worrisome finding is that in a recent developmental study researchers report that they were unable to localize the tail of the caudate in 22% of a set of high resolution MR scans they examined (Nabavizadeh and Vossough, 2013). It is unclear whether the structure will prove to be easier to localize in adults, or if improved scanning methods can practically be developed that will allow for better localization.

### Non-human animals: electrophysiological studies

Until recently, only a few studies have targeted the tail of the caudate in animal research studies. In some early monkey studies researchers found activity in response to visual stimuli. Caan et al. (1984) found that tail of the caudate and posterior putamen cells responded to a variety of complex visual stimuli. Tail of the caudate neurons were also found to be active during visual discrimination learning (Brown et al., 1995).

In the last few years, the Hikosaka lab has begun a systematic investigation of the role of the tail of the caudate in learning to make saccades to visual stimuli (Hikosaka et al., 2006, 2013). They found that neurons in the tail of the caudate code for eye movements on the basis of both stimulus identity and location (Yamamoto et al., 2012). Tail of the caudate cells were also sensitive to value of the visual stimulus, with higher activity for stimuli associated with greater reward. This pattern of activity transferred into a free viewing task including multiple stimuli: monkeys looked at previously rewarded stimuli for longer than non-rewarded stimuli even though no gaze contingent rewards were given (Yamamoto et al., 2013). Activity patterns in the SNr, which receives input from the tail of the caudate and projects to the superior colliculus to elicit saccades, were the opposite pattern, consistent with the inhibitory GABAergic projections from caudate to SNr to superior colliculus (Yasuda et al., 2012). Finally, the tail of the caudate differed from the head of the caudate in that neurons in the tail were sensitive to stable long-term value of stimuli, whereas those in the head flexibly adapted to changes in stimulus value (Kim and Hikosaka, 2013); activity patterns in the body of the caudate were intermediate. They further verified that the tail of the caudate played a causal role in saccades to stable value stimuli by inactivating it with muscimol, which selectively impaired responses to stable value stimuli.

### Lesion studies

Lesion work is challenging for a number of reasons, and as a result no specific lesion studies targeting only the tail of the caudate have been performed in non-human animals, and no human cases of brain damage limited to the tail of the caudate have been reported. However, there have been a number of isolated yet intriguing findings that imply the basal ganglia play a role in visual processing. For example, one study of premature infants found that basal ganglia damage was associated with visual impairment to a greater degree than occipital lobe damage (Mercuri et al., 1997). Section Tail of the Caudate and Amnesia discusses research that has examined the learning and memory consequences of medial temporal lobe damage, usually in the context of examining global amnesia. Section Basal Ganglia Disorders discusses what is known about visual processing deficits in the primary basal ganglia disorders, Parkinson's disease and Huntington's disease.

#### Tail of the caudate and amnesia

The only animal lesion study to specifically make a claim about the role of the tail of the caudate was a study by Teng et al. (2000). They compared monkeys with combined hippocampal and tail of the caudate lesions with lesions limited to the hippocampus and found that only the monkeys in which the tail of the caudate was lesioned were impaired on visual discrimination learning.

Given how close the tail of the caudate is to the hippocampus, the tail of the caudate may also have been damaged in some reported human cases of amnesia. The two most common etiologies of amnesia affecting the medial temporal lobe are herpes simplex encephalitis and anoxic damage. Herpes simplex encephalitis typically damages not only the hippocampus but adjacent temporal cortical regions and the amygdala (Kapur et al., 1994). Although anoxic injury is often thought to be selective for the hippocampus, there is evidence that the basal ganglia are also commonly damaged by anoxic injury (Caine and Watson, 2000; Hopkins and Bigler, 2012). One study found that patients with selective amnesia had damage limited to the hippocampus after anoxia (Di Paola et al., 2008), but not all published studies have been so careful in linking structure and function.

If the tail of the caudate is potentially damaged in amnesia, are there particular deficits currently thought to be due to damage to the hippocampus and/or medial temporal lobe cortex that may instead be due to tail of the caudate damage? The Teng et al. (2000) study described above found that impairments in visual discrimination learning were due to damage to the tail of the caudate rather than the hippocampus. Their task required animals to learn to discriminate between similar visual stimuli across multiple trials, via trial and error. This task is similar to human visual categorization tasks known to recruit the body and tail of the caudate (discussed in section Human Neuroimaging). Patients with amnesia have also been shown to be impaired on these tasks (Hopkins et al., 2004). However, this impairment could be because the task includes multiple demands, some of which may require hippocampus. One proposed contribution of the hippocampus to visual categorization learning that has received some empirical support is its role in processing novel visual stimuli, potentially in order to establish new memory traces that can then interact with the striatal learning processes (Meeter et al., 2008; Seger et al., 2011). Another is that the hippocampus can represent stimuli that are exceptions to the overall rule (Davis et al., 2012).

Patients with medial temporal lobe damage have also been reported to have other abnormalities in visual learning and memory. These are usually attributed to the specific computational functions that the damaged regions are thought to perform. Within the medial temporal lobe, multiple neocortical regions converge on the medial temporal cortex (including entorhinal, perirhinal, and posterior parahippocampal regions). From the medial temporal lobe cortex information passes through the dentate-hippocampal loop which implement functions including pattern separation and pattern completion that allow for the formation of new relational memories (Rolls, 2010; Jones and McHugh, 2011). In particular, the pattern separation functions of the dentate gyrus are important for being able to distinguish between very similar items across categories (LaRocque et al., 2013) and thus damage to the hippocampus leads to problems in forming new visual relational memories. The medial temporal cortex, in particular the perirhinal cortex, is thought to have an important role in visual memory; it is often linked to recognition memory for visual objects (Balderas et al., 2013). Some theories argue that this region should be thought of as a higher order visual processing region (Pagan et al., 2013), and have shown that people with damage to this region have problems with perceptual categorization and learning (Graham et al., 2006; Barense et al., 2012; Erez et al., 2013).

#### Basal ganglia disorders

Although no human lesions specific to the tail of the caudate have been reported, many studies of degenerative diseases that affect the basal ganglia in general have been performed. Currently little is known about how different diseases affect higher order visual processing, and if these effects are due to damage to the visual loop in particular or could be caused by damage to other loops. Patients with two of the major basal ganglia disorders, Parkinson disease and Huntington disease, are impaired in visual categorization learning (Knowlton et al., 1996; Shohamy et al., 2008) which relies on multiple corticostriatal loops including the visual loop (Seger, 2008), but it is unclear whether their impairments are specifically due to visual loop damage rather than impairments in other cognitive functions subserved by other corticostriatal loops such as feedback processing (Shohamy et al., 2008; Holl et al., 2012) or attentional shifting (Moustafa and Gluck, 2010).

Although both Parkinson and Huntington patients are impaired on visual categorization learning, the two disorders have very different underlying pathologies, different patterns of progression, and may affect the visual loop differently. In Parkinson disease there is some evidence that the visual loop should be affected relatively late because initial dopamine loss is primarily in rostral and lateral portions of the dopaminergic midbrain (Damier et al., 1999), which leads to dopamine depletion in the putamen (Kish et al., 1988). As the disease progresses it affects the anterior striatum, with the ventral striatum affected last. This pattern is reflected in shifts of functional connectivity in Parkinson's disease, with the motor loop the most strongly affected (Helmich et al., 2010). It is unclear when the tail of the caudate is primarily affected, though given overall patterns of connectivity it is most likely in parallel with the anterior striatum. In Parkinson disease the most unusual visual processing disturbance is the presence of visual hallucinations; approximately one third of the patients surveyed in one study reported visual hallucinations, generally images of animals or people that lasted for on the average for 5 min (Davidsdottir et al., 2005). Meppelink et al. (2009) found that visual hallucinations were associated with reduced object processing in higher order visual cortex and reduced bottom-up input to the prefrontal cortex. In addition, Parkinson disease patients also have some deficits in eye movement and attentional control, though these could be due to oculomotor loop or other dysfunction (Chambers and Prescott, 2010; Archibald et al., 2013).

In Huntington's disease cell loss proceeds from dorso-medial to ventro-lateral regions, with the tail of the caudate (along with medial head of the caudate dorsal putamen) having the greatest cell loss (Aylward et al., 2004). However, it should be noted that by the time Huntington disease is manifested overall damage to the basal ganglia is severe, and subsequent progression of the disease may be primarily due to increasing damage to cortex, white matter, and other subcortical structures (Georgiou-Karistianis et al., 2013). Overall, greater visual processing impairments have been reported in Huntington disease than Parkinson's disease, but typically only for difficult tasks, which leaves open the possibility that as in probabilistic classification the impairment is due to other cognitive processes besides visual processing. Gómez-Tortosa et al. (1996) found that visual deficits developed in parallel with other cognitive deficits, with early disease patients impaired only in a task involving complex visual integration. Lawrence et al. (2000) examined performance on a series of visuospatial and visual object perception tasks. Perception was unimpaired except for a very difficult object decision task (identifying degraded objects). There have been reports of deficits in recognition of emotional facial expressions, but again that could be due to known problems in processing emotion rather than problems in visual processing (Snowden et al., 2008). Several studies have found impairment on tasks that required working memory and/or recognition memory, including maintenance of individual patterns and in a delayed match to sample task These tasks all demand incorporation of visual perceptual information with selective behavioral choice, which could be dependent on the visual loop or on other corticostriatal loops (Mohr et al., 1991; Lawrence et al., 2000; Dumas et al., 2012).

## Potential functions of the visual corticostriatal loop

As described in section Anatomy of the Visual Corticostriatal System, the visual corticostriatal loop has the same circuitry as other corticostriatal loops, with the possible exception of the lack of a hyperdirect pathway. Therefore, the computational functions carried out in the visual loop should be similar to those in other corticostriatal loops, though these computations may be applied to achieve different ends. For example, the basic selection function of corticostriatal circuitry in the motor loop is used to select specific motor programs, whereas in the executive systems it contributes to working memory and cognitive strategy selection.

### Insights from computational models

A good place to start then is to consider what are the functions of the basal ganglia in the better studied motor and executive loops. Computational models of these loops generally implement one or two of the following basic mechanisms. The first is a selection (sometimes termed gating or thresholding) function. Multiple representations exist at the cortical level. As described in more detail in section Pathways Through the Basal Ganglia Output Nuclei, the basal ganglia overall exert a strong inhibition onto the thalamus, which prevents activity in the excitatory projections from thalamus to cortex. The direct pathway within the basal ganglia selects or disinhibits the representation that is most suitable for the current situation. The indirect and hyperdirect pathways modulate inhibition across varying time scales (Mink, 1996; Frank, 2005; Humphries et al., 2006).

The second is a reinforcement learning function. The basal ganglia can learn to strengthen or weaken this selection process via dopaminergic input, in a manner consistent with reinforcement learning algorithms (Lee et al., 2012; Morita et al., 2012). Most models focus on selection and reinforcement learning within a single corticostriatal loop, and incorporate very simple representations of single regions of cortex, such as a motor cortex with two potential responses (Frank, 2005, 2011; Humphries et al., 2006). These models suggest potential ways to address the closed loop function of the visual corticostriatal loop, but open loop functions require more complex cortical representations that incorporate at least two cortical regions.

With multiple cortical regions, it is possible to consider different applications of the selection process such that selection is directed to another cortical region, or affects how input from another cortical region is processed in the target region. These applications of selection are often referred to as “gating” or “routing” models. Two good examples are the FROST model developed by Ashby et al. (2005), and the PBWM (Prefrontal basal ganglia working memory) model developed by O'Reilly and colleagues (Hazy et al., 2006, 2007). PBWM model includes multiple prefrontal regions or “stripes” that maintain and update working memory. Updating happens when the direct pathway disinhibits a prefrontal stripe and allows a new item to enter into working memory; this process is referred to as gating. Perceptual items are represented in a separate cortical module, which both projects directly to the PFC region, and to the striatum as well. Because the focus of this model is the basal ganglia and prefrontal cortex components, perceptual information is represented in highly abstracted form (e.g., in terms of fully determined features and/or object identity).

Stocco et al. (2010) take an alternate approach, which they term “routing,” in which interaction between multiple cortical regions is through closed loop mechanisms plus cortico-cortical input from other regions. In their model the cortical portion of the corticostriatal loop receives broad inputs from other cortical regions. The closed loop functions to select or inhibit these inputs through the direct and indirect pathways.

### Possible function of recurrent (closed loop) projections in the visual loop

As summarized above, and illustrated in Figure 4, the main evidence for recurrent connections to temporal cortex in primates is the Middleton and Strick (1996) study finding a closed loop through temporal area TE. It is possible that TE is atypical, and most temporal regions don't have recurrent connections. This is not unprecedented: in the corticocerebellar system most cortical regions project to the cerebellum, but not all receive closed loop return projections (Bostan et al., 2013). Furthermore, anatomical connectivity is a necessary prerequisite, but is not sufficient. The brain has a plethora of connections between disparate regions, many of which are usually weak or dormant. One example is that the occipital lobe receives projections from auditory and somatosensory regions, but does not show significant sensitivity to these sensory modalities in sighted persons. However, in the blind these projections can strengthen and allow for the occipital lobe to be recruited for tactile and/or auditory processing (Pascual-Leone et al., 2005).

Assuming for the moment that there are robust and active closed loop recurrent projections to the visual cortex, what might be their function? This function should be consistent with what we know about the computations identified in the other corticostriatal loops, as summarized above, namely selection via the direct route, and modulation of inhibition through the indirect route. What use might selection and inhibition be in higher order visual processing? The most direct application of the concept of selection is that these connections would inhibit alternative representations of visual information, and select the dominant one for further processing. The visual cortex does have to resolve ambiguity (the input to the visual system is often consistent with many potential interpretations and can be parsed in many ways), and this mechanism is at least theoretically consistent with our knowledge of basal ganglia function. There are also cortico-cortico projections between visual regions that may provide inputs that are subject to these selection processes.

There has been little research investigating basal ganglia activity in conditions of visual ambiguity. The strongest evidence for a causal role of the visual corticostriatal system in this process would come from lesion studies in primates or studies of basal ganglia disorders in humans. As described above in section Basal Ganglia Disorders, visual symptoms of basal ganglia disorders have not been widely studied, and it is unclear whether the impairments that have been reported could be due to problems in resolving visual ambiguity.

### Function of open loop projections in the visual loop

Open loop models involve effects on other brain structures. As discussed in section Open Loops from the Visual Corticostriatal System, interactions between corticostriatal loops often follow the gradient within the striatum from motivational loop, through to the associative loops (including the visual loop), to the motor loop. Therefore, with respect to the visual loop, it makes sense to focus on potential open loop projections to the cortical regions participating in the associative and the motor loop, and how the selection or gating function of the striatum might be utilized in each cortical region. Largely, these regions are the frontoparietal networks underlying a hierarchy of executive control including both cognitive and motor functions (Badre, 2008), and selection may be utilized for motor or cognitive functions. This section discusses three potential frontoparietal network open loop targets. The first is open loop projections to premotor regions to enable behavioral choice. The second is projections to oculomotor networks enabling shifts in visual attention and eye movements. Control of eye movements allows for better perception (as items are foveated), and also the ability to ultimately make decisions about the visual stimulus and subsequently choose an appropriate course of action. Finally, open loop projections from the visual loop to executive regions such as the lateral prefrontal cortex may allow for visual information to be maintained or manipulated in working memory during extended cognitive processing, rather than being immediately used to select a motor or eye movement response.

#### Visual conditional response performance and learning

A logical extension of the idea that the basal ganglia are important for selection of motor programs is the idea that the non-motor loops ultimately have the function of interacting with the motor region to allow the organism to learn to select and execute motor responses that are appropriate to the current situation. The basal ganglia are involved in a variety of tasks in which subjects learn to perform a conditional response on the basis of a stimulus or situation (Seger, 2008, 2009), including arbitrary visuomotor association learning (Wise and Murray, 2000), category learning (Seger and Miller, 2010), habit learning (Yin and Knowlton, 2006; Graybiel, 2008), and decision making (Summerfield and Tsetsos, 2012; Seger and Peterson, 2013). These tasks all have in common a trial structure in which the subject is presented with a stimulus or cue, most often visually, makes a response conditional on the cue, then receives feedback or reward if the response was correct. Studies indicate that the striatum as a whole makes several contributions to category learning, resulting in recruitment of different striatal regions during different portions of a trial. For example, the putamen is most active when making the motor response, and the head of the caudate is most active when processing the stimulus and receiving feedback (Peterson and Seger, 2013).

Studies examining the tail of the caudate (summarized in section Human Neuroimaging and Figure 6) find that it is active during stimulus processing, consistent with it playing a role in visual processing for categorization. Conditional responses to visual stimuli could be supported by open loop projections from the visual loop to motor structures. One example described above in section Non-human Animals: Electrophysiological Studies is the work by Hikosaka and colleagues investigating the open loop direct projection to the superior colliculus which allows for eye movements to be sensitive to the learned value of the visual stimuli. Another example is visual categorization, in which visual stimuli provide the information to choose the appropriate category and motor response used to indicate the category. The COVIS model proposed by Ashby et al. (1998, 2007) models visual categorization through a direct open loop projection from the visual loop to the pre-SMA, consistent with known output projections from the VAmc region of the thalamus that participates in the visual loop.

However, it is possible that the role of the visual loop in conditional learning is indirect rather than via direct projections to motor regions. Anatomically, the pre-SMA is strongly interconnected with prefrontal regions, and does not directly project to SMA and other premotor regions (Picard and Strick, 1996). Instead of directly selecting motor responses, the visual loop projections to this region could serve to select more abstract categorical representations, which then contribute to motor response selection via projections from prefrontal to premotor regions. Recent work by the Ashby lab (Waldschmidt and Ashby, 2011) indicates that direct motor selection of categorical responses may be accomplished through the motor loop involving the putamen, possibly via the known cortricostriatal projections from parietal lobe regions to the putamen. Overall, studies find a shift in networks during conditional response learning from executive to motor, and the visual loop may more strongly affect learning in the early stages in interaction with executive regions. This shift from associative to motor loops is consistent with a large body of research in rodents finding a shift from dorsomedial striatum (homolog of the anterior caudate) to dorsolateral striatum (homolog of the putamen) as learning progresses from being goal-directed to habitual (Balleine et al., 2009).

#### Visual attention and eye movement control

Another possible target of open loop projections from the visual loop are frontoparietal regions involved in visual attention and eye movement control, including the frontal eye fields and parietal cortex. These regions interact direct with another region of the caudate, the lateral body, in the oculomotor loop. Several recent reviews have considered the anatomy of this system and its function in regulating eye movements (McHaffie et al., 2005; Shires et al., 2010). Recent theories have argued that this system is important for visual attention more broadly. Visual attention involves collecting and integrating sensory and cognitive data about the world in order to focus processing on potentially important objects and their spatial locations. Perceptual and motor factors in visual attention are tightly connected, and that as a result this system allows for guiding action to objects and locations, in particular eye movements (Gottlieb and Balan, 2010). Within this network, area LIP (located around the Intraparietal sulcus in humans) is often considered to represent a spatial salience or priority map (Bisley and Goldberg, 2010) in which spatial location is combined with other important information about objects including reward value, category membership, amount of information supporting a particular perceptual decision, etc. The oculomotor loop caudate neurons in this region are sensitive to many of the same factors as LIP and FEF (Watanabe and Munoz, 2013). Ding and Gold (2010, 2012, 2013) found that multiple relevant variables for perceptual decision making were coded for in the body of the caudate, including cells sensitive to information accumulation, decision threshold, and bias before actual stimulus toward a left or right saccade. Harsay et al. (2011) have examined the system in humans, and found that functional connectivity between the cortical oculomotor regions and caudate predicts learning in a saccade task.

The evidence for the importance of both visual loop and oculomotor loop processing in controlling eye movements raises the question of how the visual loop might interact with the oculomotor loop through open loop projections. One known open loop projection from the visual loop is the preSMA, which is adjacent to the FEF and considered along with FEF to fall within Brodmann's area 8. In addition, interaction between loops could be through cortical regions; there are known anatomical and functional connections between LIP and temporal lobe visual processing regions (Gold and Shadlen, 2007).

#### Visual working memory

Another possibility is that open loop projections are important for visual working memory, in particular selecting or gating which visual representations should be maintained and processed in working memory. This interpretation brings together two strands of research in working memory: the first one focusing on cortex and showing that working memory for visual items (e.g., objects or faces) involves interaction between frontoparietal working memory systems and the temporal lobe regions important for representing those items (Clapp et al., 2010; Gazzaley and Nobre, 2012). The second is research showing that the striatum is important for selecting what items should be gated into working memory. Several theories of gating emphasize open loop projections in which the selected items are gated as representations in higher order systems, but may also require recurrent projections combined with cortico-cortico projections.

There have been a large number of studies finding a role of the basal ganglia in working memory, but most of them have focused on the executive function components involving frontoparietal and anterior striatum interactions to maintain, select and update working memory. Most empirical work has supported the idea that the basal ganglia are especially important for selecting which items should enter working memory, often by filtering the possible inputs (McNab and Klingberg, 2008; Baier et al., 2010).

## Conclusion and future directions

In summary, there is substantial anatomical and functional evidence for a visual loop through the tail of the caudate nucleus. The visual loop however, has received much less attention from researchers than loops through the frontal cortex supporting executive, motor, and motivational functions. One goal of this review is to encourage basal ganglia researchers to consider how the visual loop might interact with other basal ganglia systems that they study. Another goal is to highlight important future directions of research in this area.

There are many ways in which our knowledge of the anatomy of the visual corticostriatal loop is limited which could fruitfully be addressed in future research. Our knowledge of the projections from cortex to striatum is based on a small number of studies in monkeys, and it is still unknown exactly which visual regions project to which striatal regions in humans (section Projections from Visual Cortex to the Caudate Tail). Although there appears to be no visual hyperdirect pathway in primates, the data available is not conclusive (section Pathways Through the Basal Ganglia Output Nuclei). Our knowledge of recurrent closed loop projections from temporal lobe is based on only one published study examining a single temporal region in monkey; although it is a plausible assumption that other visual cortical regions form similar closed loops, it has not been verified empirically (section Closing the Loop: Projections to Thalamus and Back to Cortex). Finally, we do not have complete knowledge of all the potential targets of open loop connections from the visual loop (section Open Loops from the Visual Corticostriatal System).

Empirical studies of the tail of the caudate have been hampered by methodological limitations. In neuroimaging, future research should focus on development of new high resolution scanning and spatial normalization processes to allow identification of the tail of the caudate on an individual subject level and support group analyses across subjects (section Human Neuroimaging). Researchers studying medial temporal lobe damage should develop ways to assess whether tail of the caudate damage has occurred in amnesic patients, and to distinguish between behavioral impairments due to tail of the caudate damage and those due to damage to adjoining structures (section Tail of the Caudate and Amnesia). Finally, researcher studying basal ganglia disorders should consider the potential effects of damage to the tail of the caudate and avoid an exclusive focus on motor and executive functions (section Basal Ganglia Disorders).

The fundamental functions of the visual corticostriatal loop are still unknown. No well-developed theories have addressed the role of recurrent closed-loop projects back to visual cortex (section Possible Function of Recurrent (Closed Loop) Projections in the Visual Loop). Several theories propose specific functions for some of the open loop projections from the visual loop, but because these projections have not been fully characterized anatomically, we do not yet have a full picture of their functions (section Function of Open Loop Projections in the Visual Loop). This paper suggested a number of possible closed and open loop functions of the visual corticostriatal loop, but developing and testing complete theories awaits future research.

## Author contributions

Carol A. Seger determined the content and wrote the paper.

### Conflict of interest statement

The author declares that the research was conducted in the absence of any commercial or financial relationships that could be construed as a potential conflict of interest.
